# Physical education course satisfaction scale for Chinese college students: development and psychometric properties

**DOI:** 10.3389/fpubh.2025.1717552

**Published:** 2026-01-16

**Authors:** Zhengcheng Jin, Chuanhao Xi

**Affiliations:** 1 School of Physical Education, Chongqing University of Posts and Telecommunications, Chongqing, China; 2 College of Physical Education, China West Normal University, Nanchong, China

**Keywords:** CFA, EFA, measurement invariance, physical education course, satisfaction assessment tools

## Abstract

**Objective:**

The scientific community still lacks effective assessment tools for evaluating college students’ PE course satisfaction. Addressing this gap will help to foster improved learning experiences and satisfaction among college students during PE course, and help achieve better health outcomes and support consistent healthy habits. Accordingly, this study aims to develop a PE course satisfaction scale for Chinese college students (PECSS-CCS).

**Methods:**

We identified a primary scale based on a literature review and semi-structured interviews with experts. This scale were then administered to 996 Chinese students from colleges within a public university. The exploratory factor analysis (EFA) and confirmatory factor analysis (CFA) were combined to establish and verify the structural validity of PECSS-CCS. The scale’s reliability was assessed through internal consistency and temporal stability tests. To assess the validity of the scale, we administered the tests of content validity, construct validity, convergent validity and criterion-related validity. And we verified measurement invariance across gender groups.

**Results:**

According to EFA (*N* = 404), we extracted six factors namely teaching characteristics, teacher characteristics and attitudes, course experience, learning atmosphere, facilities and examination. The CFA (*N* = 472) fit indices results were χ^2^/dƒ = 1.705, RMSEA = 0.039, CFI = 0.968, TLI = 0.963 and SRMR = 0.042. For the reliability, the Cronbach’s alpha coefficient ranged from 0.827 to 0.924, the McDonald’s omega value was 0.833 to 0.925, the composite reliability (CR) values ranged between 0.841 and 0.96, while the split-half reliability coefficient was 0.862, and the test–retest coefficient was 0.752 to 0.884 (*N* = 120). For the validity, the average variance explained (AVE) value ranged from 0.575 to 0.702, the PECSS-CCS subscales were positively correlated with basic psychological needs in exercise scale (BPNES), the correlation coefficient was 0.320 to 0.554. For the measurement invariance, the ΔCFI, ΔSRMR, and ΔRMSEA were all under 0.010. Therefore, the main psychometric indicators of the scale had reached the satisfactory statistical standard.

**Conclusion:**

In summary, the PECSS-CCS, which includes 26 items, demonstrates satisfactory reliability and validity, serving as a valid assessment tool for PE course satisfaction among Chinese college students.

## Introduction

Continuous decline in health behavior among college and university students has increased over the years (1–3), it has become a major public health issue worldwide and China is no exception (4, 5). According to recent official reports released by the Chinese Sports, Health, and Art Education Department, the physical health level of Chinese college students has shown a significant decline, with physical fitness test failure rates reaching 30% (6). Declining physical health among Chinese college students has emerged as a critical issue within higher education. The decline in physical health is not limited to reduced physical fitness, but is also accompanied by sedentary lifestyles, psychological maladjustment, and weakened engagement in meaningful activities (7). Empirical evidence further suggests that physical exercise can indirectly improve college students’ mental health (8). Moreover, a recent empirical study has demonstrated that physical exercise affects health outcomes through identifiable psychological mechanisms, including resilience, cognitive regulation, and emotional well-being (9). Physical education (PE) is a crucial factor in promoting young people’s participation in physical activities (10). Furthermore, PE is a significant part of improving the physical condition of college students (11) and promoting a positive lifestyle (12). This emphasizes the importance of exploring ways to improve the quality of PE courses in colleges.

Some scholars believe that satisfaction with PE courses is closely connected with the quality of the course; for example, better satisfaction during PE courses results in stronger exercise adherence (13). Additionally, compared with exercise adherence, satisfaction had a greater impact on exercise tendencies (14). The satisfaction scale effectively assesses student satisfaction with PE courses (15). However, previous scales have been designed to assess the basic psychological need satisfaction obtained from PE (16–18) and few studies have reported PE course satisfaction scales for college students based on comprehensive psychometric validation procedures. Given the critical role of student satisfaction in PE courses and existing gaps in validated measurement instruments, we developed and validated a PE course satisfaction scale to assess Chinese college students’ satisfaction with PE courses and improve the understanding of college students’ needs and expectations in PE courses. Before filling this gap, what factors should be considered to form a PE course satisfaction scale? Some previous studies on PE and PE satisfaction provide some inspiration.

First, the quality of physical education instruction has gained significant attention in recent years (19), and teachers’ knowledge and skills are crucial factors in improving the quality of PE (20, 21). Practice-based teacher education emphasizes that a teacher should master sufficient knowledge and skills before teaching a PE lesson (22). Therefore, PE teachers significantly influence PE learning (23) and could help encourage students’ autonomy and improve their concentration (24). Simultaneously, PE teachers with certain knowledge and competencies could help students with their movement capability (25). Furthermore, teacher strategies in maintaining discipline could influence students’ satisfaction with the PE curriculum (26); however, teachers’ overly controlling behavior negatively affect the mental experiences of students in PE class (27). Therefore, we propose that the teacher characteristics and attitudes is an important factor in PE instruction and student satisfaction.

Subsequently, previous research indicates that campuses cannot develop sustainably without physical facilities (28) and facilities may influence students’ experience, attitude, and satisfaction (29–31). Meanwhile, PE courses also require high-quality equipment and facilities (32). In addition to the increased distance of sports facilities from residential areas that may reduce the willingness of students to exercise, the lack of facilities is a conceivable obstacle for physical activity programs (33–35). Facilities seem to be essential for students to achieve their recommended physical activity (36). Therefore, facilities are another key factor for effective PE instruction satisfaction.

Third, PE courses’ examinations can test students’ motor skill competency (37) and are an important element of PE classes (38). The use of fitness exams in PE has been heavily discussed in PE research (39). For example, students’ fitness examinations can promote a positive exercise lifestyle (40)and has long-term health benefits (41). Moreover, motor skill test results provide valuable data for planning and structuring physical education and sports training programs (42). Therefore, we consider examination as an essential factor in PE satisfaction.

Additionally, former studies show that a positive learning atmosphere is effective for students (43, 44). For instance, a funny atmosphere could result in positive attitudes among students (45). Conversely, a serious atmosphere in the classroom can make students feel stressed (46); accordingly, teachers should create a positive educational atmosphere to achieve quality learning (47). Moreover, the learning atmosphere positively influences students’ satisfaction and motivation (48, 49). Because a conducive learning atmosphere can effect higher learning satisfaction (50), we determined that a learning atmosphere could be a vital factor in PE instruction satisfaction.

Furthermore, previous studies suggest that a need-supporting environment is positively related to satisfaction in the context of PE (51) and being ignored by teachers can result in negative experiences in PE class (52). In contrast, having fun, developing motor skills and taking challenges can result in meaningful experiences to students during PE courses (53). Students’ satisfying experience will positively influence their satisfaction in physical activity (54). Therefore, course experience might contribute to PE course satisfaction.

Lastly, we inferred the existence of teaching characteristics because appropriate teaching content, innovative teaching method, effective schedule and other teaching characteristics in PE were frequently discussed in curriculum research, especially in the Chinese case (55–57).

### Research questions and hypotheses

Based on the scale development and validation, the present study proposed one research question: what underlying factors constitute physical education course satisfaction among Chinese college students, and can a psychometrically sound satisfaction scale be developed and validated based on these factors? Subsequently, the following hypotheses were formulated to address the research question:

*H1:* The Physical Education Course Satisfaction Scale for Chinese College Students (PECSS-CCS) is hypothesized to exhibit a stable six-factor structure, comprising teaching characteristics, teacher characteristics and attitudes, course experience, learning atmosphere, facilities, and examination.

*H2:* The PECSS-CCS is hypothesized to demonstrate satisfactory reliability, including adequate internal consistency and temporal stability over time.

*H3:* The six-factor model of the PECSS-CCS is hypothesized to demonstrate adequate construct validity, as evidenced by acceptable model fit indices and convergent validity indicators.

*H4:* Scores on the PECSS-CCS are hypothesized to be significantly associated with external criterion variables related to physical education learning outcomes, supporting criterion-related validity.

*H5:* The PECSS-CCS is hypothesized to demonstrate measurement invariance across gender groups, indicating that the scale assesses physical education course satisfaction equivalently for male and female college students.

## Materials and methods

### Methods

The research instrument was developed through three stages: (1) dimensions delimitation and items generation; (2) items purification and validation; and (3) test administration, psychometric properties test and scale optimization.

### Phase 1: Dimensions delimitation and items generation

To explore the initial dimensional framework, a comprehensive literature review and semi-structured expert interviews were conducted. The expert panel comprised five specialized researchers (58), including: two full professors with PE expertise; two associate professors possessing over fifteen years of PE teaching experience; and one associate professor with extensive experience in PE course design. This panel was engaged to primarily capture and define the facets of college students’ satisfaction with PE courses based on the extant literature and their experience. Therefore, we have preliminary proposed six dimensions of the PE course satisfaction scale for Chinese college students: teaching characteristics, teacher characteristics and attitudes, course experience, learning atmosphere, facilities and examination (see Table 1).

**Table 1:** Definitions of six dimensions of PECSS-CCS.

Dimension	Definition
Teaching characteristics	This dimension represents students’ satisfaction with the components that make up the PE teaching characteristics.
Teacher characteristics and attitudes	This dimension represents students’ satisfaction with the image, temperament, spiritual qualities, and teaching skills of their PE teachers.
Course experience	This dimension represents students’ satisfaction with their feelings of learning and improvement when participating in a PE class.
Learning atmosphere	This dimension represents students’ satisfaction with the demographic characteristics and peer relationships in PE class.
Facilities	This dimension represents students’ satisfaction with the various conditions of sports equipment and venues in PE class.
Examination	This dimension represents students’ satisfaction with various aspects related to the examination of PE courses.

To generate the initial items representing the six dimensions, we conducted a comprehensive review of the literature pertaining to PE satisfaction and validated satisfaction scales. Item inspiration was derived from Shi’ s description of college students’ learning satisfaction within PE courses (59). Furthermore, validated satisfaction instruments provided additional references for compiling the initial item pool (60–64). This process yielded an initial set of 53 items. These items were written to assess PE course satisfaction specifically from the perspective of college students within the Chinese educational context.

### Phase 2: Items purification and validation

After item generation, a paper-based expert survey was employed to evaluate content validity. The expert panel involved in preliminary interviews assessed items against five criteria: (1) construct representatives, (2) domain relevance, (3) content comprehensiveness, and (4) clarity (65, 66). Items failing to meet predetermined criteria following expert review were eliminated from the item pool. Experts also categorized items into the appropriate dimensions of college students’ PE course satisfaction. Items achieving consensus (endorsed by ≥4 experts) regarding their designated dimension proceeded to subsequent validation phases. Consequently, 30 items were retained, theoretically reflecting the dimensions of college students’ PE course satisfaction: teaching characteristics (5 items), teacher characteristics and attitudes (6 items), course experience (5 items), learning atmosphere (4 items), facilities (5 items), and examination (5 items). Subsequently, a pilot study evaluated item clarity and comprehensibility. This evaluation was administered via individual semi-structured interviews. Participants identified and elaborated on linguistic ambiguities or conceptually unclear statements within the items. Items containing inappropriate expressions were revised based on participant feedback.

### Phase 3: Test administration, psychometric properties test and scale optimization

The next step was to test and select items with theoretical and psychometric properties. First, we utilized item-total correlation analysis to assess the association between individual items and the total scale score, evaluating how well each item represented the target construct. Second, we conducted an exploratory factor analysis (EFA) on the retained items to examine the scale’s factor structure. Prior to factor analysis, we assessed the item data using the Kaiser-Meyer-Olkin (KMO) measure and Bartlett’s test of sphericity. We utilized principal component analysis to reduce the dimensionality of observed variables, applying promax rotation because of the assumption of correlations among latent factors (67). Next, we employed confirmatory factor analysis (CFA) to validate the construct validity of the scale with 26 items (68). Considering the data exhibited significant deviation from multivariate normality, as evidenced by Mardia’s coefficient of 64.02 (*p* < 0.01), the robust maximum likelihood (MLR) was utilized for the estimation (69). Moreover, we conducted multiple-group analysis to test measurement invariance across gender subgroups (70). Furthermore, we calculated Cronbach’s alpha, McDonald’s omega, and composite reliability (CR) to assess internal consistency, while test–retest reliability was utilized to evaluate temporal stability. Ultimately, we calculated the average variance extracted (AVE) to evaluate convergent validity, and performed Pearson correlation analyzes to assess criterion-related validity.

### Participants

This study recruited 996 participants from colleges within a public university in Chongqing Province, China. Using a purposive sampling method, all the participants are Chinese. The participants comprised 355 freshmen, 380 sophomores and 261 juniors including 570 males and 426 females. Fewer senior students in China took the PE course; accordingly, they were not used as a sample source. Participants’ ages ranged from 18 to 22 years, with a mean of 19.22 years and a standard deviation of 0.92.

The studies involving humans were approved by Ethics Committee of Chongqing University of Posts and Telecommunications (No. 20231017), all participants were provided appropriate informed consent, volunteered for the online test, and were guaranteed that all information would remain strictly confidential and anonymous. The questionnaires were administered during normal PE class time with the assistance of the class teacher, and took 8–10 min to complete in average. All participants took PE course regularly each semester. Each PE class had 40–50 students, and each PE course lasts for 90 min. All class teachers are physical education majors and have master’s degree in education.

## Measurement and analysis procedures

### Measures

All participants provided demographic data encompassing age, gender, and current grade level. The participants consisted of three groups. Sample 1 in the first group comprising 404 participants (male = 229, female = 175) completed a 30-item PECSS-CCS. Sample 2 in the second group including 472 participants (male = 274, female = 198) completed the 26-item PECSS-CCS and the 12-item basic psychological needs in exercise scale (BPNES). Sample 3 in the third group including 120 participants (male = 67, female = 53) completed the 26-item PECSS-CCS twice during a 4-week interval. The measurement instruments underwent professional translation equivalence. All tests were written and administered in Mandarin Chinese. A separate specialist back-translated the finalized PECSS-CCS into English for publication.

#### PECSS-CCS

The initial version of PECSS-CCS has six dimensions of 30 items, and the finalized version of PECSS-CCS consists of 26 items. Both the initial scale and the finalized scales were used to test the degree of college students’ PE courses satisfaction. Responses were rated on a 5-point Likert scale (1 = Strongly disagree, 5 = Strongly agree).

#### BPNES

The 12-item BPNES (71) was used to evaluate the criterion-related validity. The BPNES was used as a means of criterion-related validating PECSS-CCS scores in this study because basic psychological needs in exercise are associated with PE course satisfaction (72). The BPNES consisted of three factors: Autonomy (4 items), Competence (4 items), Relatedness (4 items). Items were evaluated on a 5-point Likert scale, in which 1 point means “completely disagree” and 5 points means “completely agree”. Liu, Chung, and Duan reported the good validity and reliability of this questionnaire in Chinese version (73). In the current study, the Cronbach’s alpha coefficient for the subscale and the overall scale were 0.851, 0.872, 0.891 and 0.92, and the McDonald’s omega coefficient for the subscale and the overall scale were 0.854, 0.872, 0.891 and 0.92, respectively.

### Analysis procedures

First, item-total correlation analysis was performed on the data from Sample 1 using SPSS27.0. During this section, items were deleted following the namely criteria: (1) the item is significantly uncorrelated with the total score and (2) the total correlation coefficient of the item is below 0.30 (74). Second, EFA was employed on the data from Sample 1 using SPSS27.0. During this time, items were removed using the following standard: (1) factors with eigenvalues less than 1 (75), (2) factor loadings of items were less than 0.40, (3) a factor with fewer than three items (76) and (4) items with cross-loadings (77). Subsequently, CFA was conducted on the data from Sample 2 using MPLUS 8.30. During this phase, the values for the model fit are required to reach the standard as follows: (1) the ratio of chi-square to its degrees of freedom (χ2/ԁƒ) was less than 3, (2) the root of the mean square error of approximation (RMSEA) value was less than 0.08, (3) comparative fit index (CFI), Tucker–Lewis index (TLI) were all above 0.90, and (4) standardized root Mean square residual (SRMR) was less than 0.08 (78). Following this, multiple-group analysis was executed on the data from Sample 2 using MPLUS 8.30, A value of ΔCFI, ΔSRMR and ΔRMSEA smaller than or equal to −0.01 indicates support for measurement invariance (79). Then, Cronbach’s alpha and McDonald’s omega coefficient for the for the 26-item PECSS-CCS and BPNES subscales were computed using the Sample 2, a coefficient is higher than 0.70 is acceptable (80). CR values for the 26-item PECSS-CCS subscales were calculated using the Sample 2, the values for each subscales are above0.70 is satisfactory. AVE values for the 26-item PECSS-CCS subscales were evaluated using the sample 2, considering 0.50 as acceptable cut-off point (81). Pearson correlation analyzes were utilized between the subscale scores of PECSS-CCS and BPNES using the sample 2, a coefficient was higher than 0.30 was considered acceptable (82). Ultimately, Pearson correlation coefficient between the two rounds of 26-item PECSS-CCS subscales were estimated using the sample 3, the result is acceptable when values are equal to 0.70 or higher (83).

## Results

### Item-total correlation analysis

Item-total correlation analysis revealed significant correlations (*r* > 0.30) between each item and the total scale score, except the item “The composition of evaluation is reasonable” (*p* > 0.05) and “course duration is reasonable” (*r* = 0.239). Accordingly, two items were deleted in this step, 28 items were retained.

### EFA

**Figure 1:**
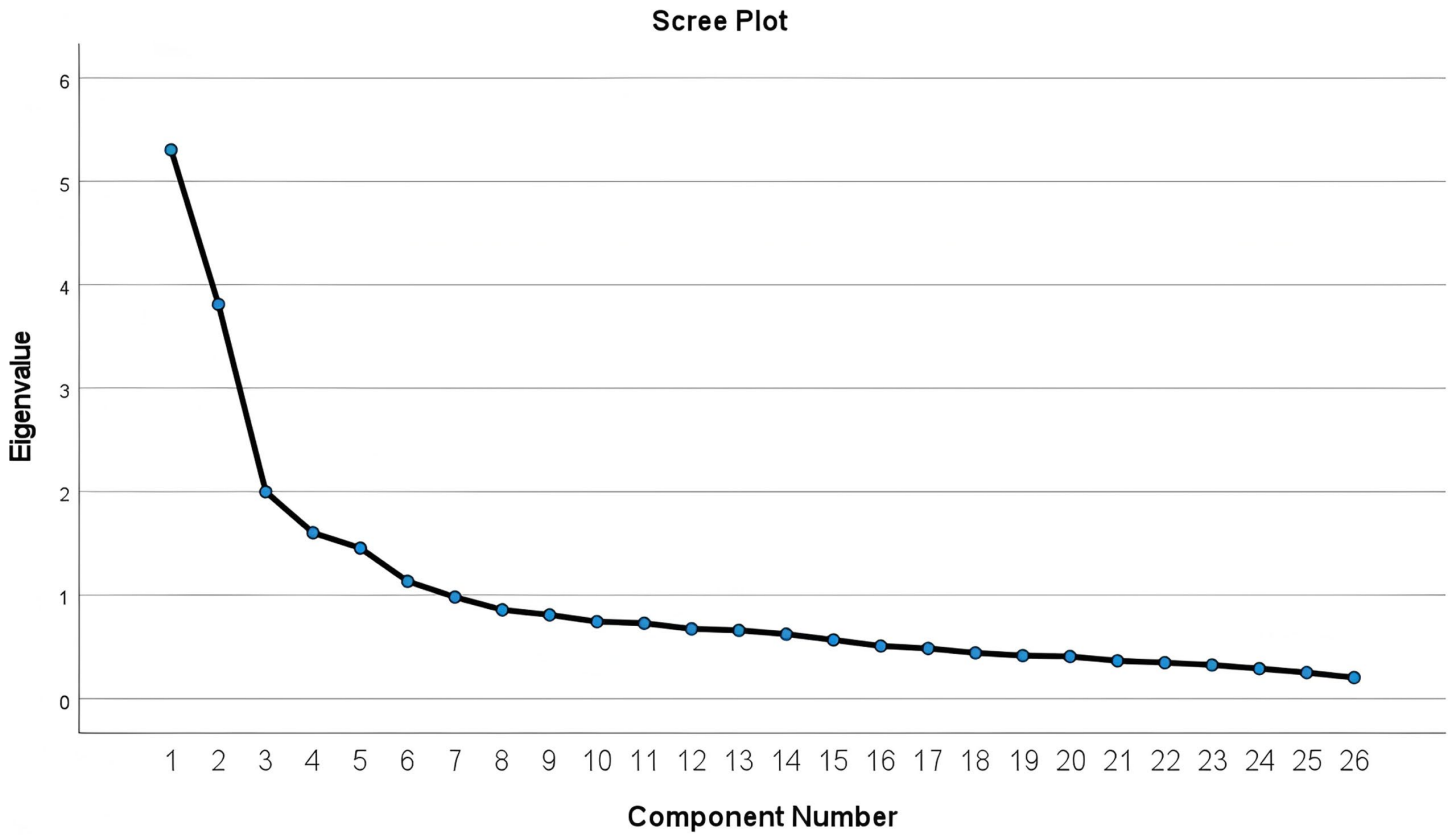
The gravel plot of the EFA.

The results of EFA indicated a KMO measure of sampling adequacy of 0.853, and Bartlett’s test of sphericity yielded a value of 3206.869, *p* = 0.000, indicating that factor analysis was appropriate. After two rotations, two items were eliminated according to statistical standards stated earlier. Finally, we identified six common factors with eigenvalues exceeding 1.0, and all retained items demonstrated factor loadings above 0.40 across their respective factors (see Table 2). The gravel diagram (Figure 1) clearly shows that the slope of the line tends to be flat after the sixth factor. Accordingly, the structure of the six factors in this study is clear. These results supported the hypothesized six-factor structure of the PECSS-CCS.

**Table 2:** Results from an EFA of PECSS-CCS.

PECSS-CCS item	Factor loadings					
	1	2	3	4	5	6
Factor 1: Teacher characteristics and attitudes						
18. The attitude of teachers is responsible	**0.842**	−0.042	0.007	−0.056	−0.101	0.033
19. Teachers have good morals	**0.687**	0.017	0.092	−0.076	−0.057	0.158
20. Teachers have good temperament	**0.668**	−0.012	−0.093	0.286	−0.018	−0.216
12. Teachers have good presentation skills	**0.636**	0.042	−0.070	0.003	0.220	−0.134
13. The teacher’s guidance is reasonable	**0.604**	−0.074	0.014	−0.186	0.228	0.104
17. Teachers have strong communication skills	**0.551**	0.055	0.071	0.072	−0.059	0.116
Factor 2: Facilities						
38. The equipment range is complete at the venue	0.017	**0.817**	−0.035	−0.018	0.115	−0.074
39. Sufficient quantity of equipment at the venue	0.017	**0.788**	−0.014	−0.142	0.148	−0.011
40. The site facilities are of good quality	−0.079	**0.769**	−0.033	−0.045	0.081	−0.027
37. The venue has good lighting condition	−0.072	**0.593**	0.143	0.195	−0.131	0.102
36. Venues are in good sanitary conditions	0.136	**0.578**	0.019	0.067	−0.200	0.212
Factor 3: Examination						
44. The exam content is reasonable	−0.018	−0.104	**0.849**	0.040	0.090	−0.041
45. The exam format is reasonable	−0.081	−0.013	**0.787**	−0.015	−0.062	0.120
43. The teacher’s evaluation is fair	0.146	0.023	**0.740**	−0.090	0.015	−0.023
47. The exam grading standard is reasonable	−0.013	0.140	**0.619**	0.114	−0.022	−0.133
Factor 4: Learning atmosphere						
32. Students would help each other	0.021	−0.132	0.063	**0.758**	−0.039	0.102
31. Students have a strong sense of teamwork	0.117	−0.077	0.091	**0.695**	0.087	−0.113
34. The class size is moderate	−0.184	0.077	−0.111	**0.669**	0.099	0.095
33. Balanced ratio of male to female students	0.021	0.082	0.010	**0.610**	0.016	−0.030
Factor 5: Course experience						
24. I can improve my motor skills	−0.010	0.066	0.079	−0.012	**0.772**	−0.001
25. I can strengthen my physical fitness	0.053	0.039	−0.054	−0.018	**0.747**	0.036
23. The exercise is effective	0.029	0.134	0.039	0.168	**0.608**	−0.043
26. I can learn new knowledge	0.014	−0.162	−0.080	0.187	**0.457**	0.351
Factor 6: Teaching characteristics						
1. The teaching content is good	−0.057	−0.008	−0.001	0.024	0.063	**0.804**
2. The teaching methodology is innovative	0.162	0.106	−0.154	0.088	−0.145	**0.652**
7. The teaching schedule is reasonable	−0.050	−0.013	0.123	−0.090	0.202	**0.642**
Eigenvalues	6.526	2.079	1.791	1.476	1.402	1.169
Explained variance (%)	25.100	7.997	6.888	5.677	5.394	4.496

*N* = 404. The extraction method was principal component analysis (promax with Kaiser normalization). Factor loadings above 0.40 are in bold. Rotation converged in 6 iterations.

### CFA

**Figure 2:**
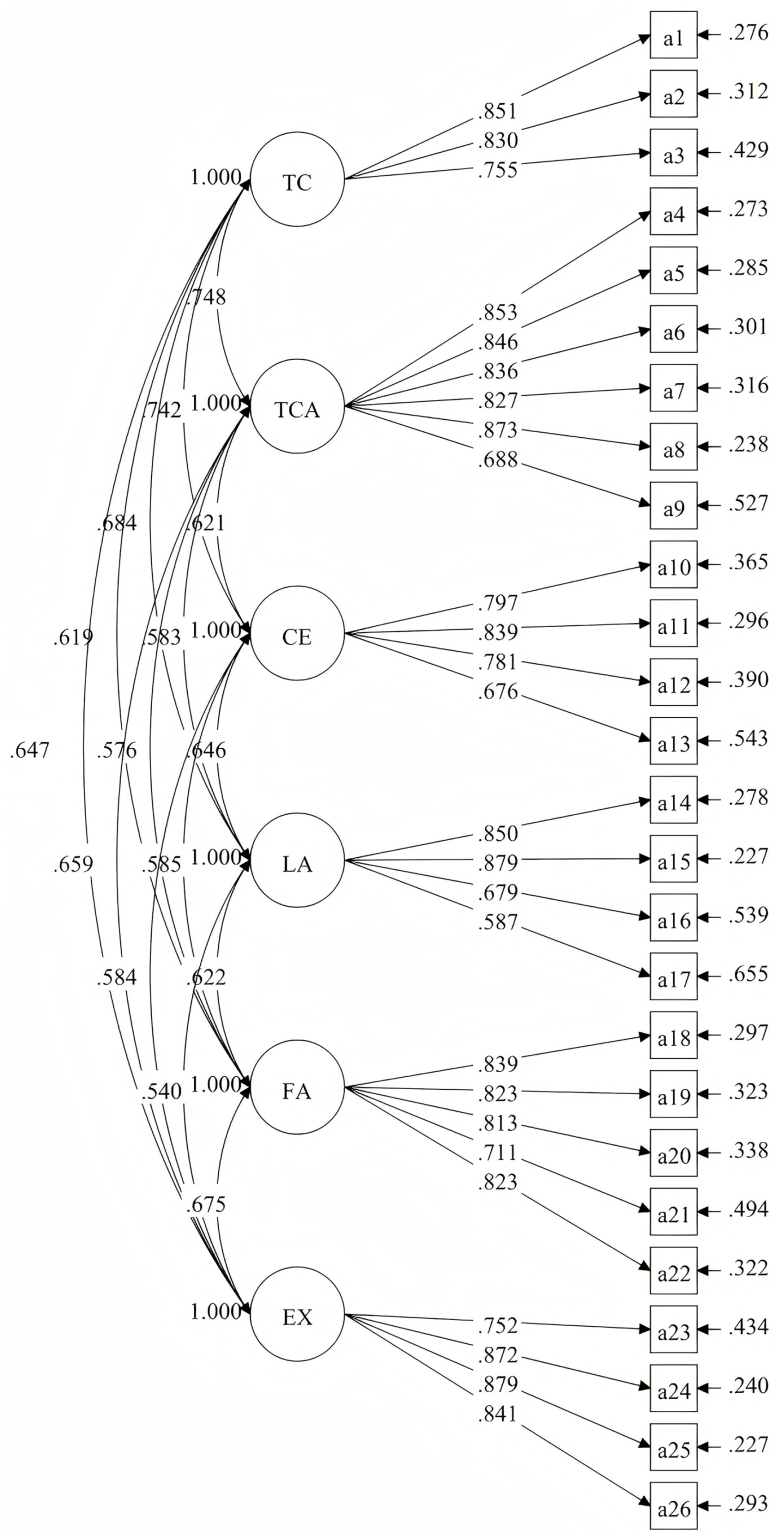
The path diagram of standardized parameters estimation of CFA.

Results from the CFA include, χ2 = 484.436, ԁƒ = 284, χ2/ԁƒ = 1.706. RMSEA = 0.039. The CFI = 0.968, TLI = 0.963 and SRMR = 0.042, all of which reached acceptable statistical standards. Therefore, the structural model in this study had a high degree of model fit and good construct validity. Additionally, all items demonstrated factor loadings above 0.50 in their respective dimensions, confirming that each item was adequately captured by its latent construct and exhibited substantial explanatory power (see Figure 2). Taken together, the CFA results and AVE values provided empirical support for the construct validity of the PECSS-CCS.

### Measurement invariance for gender

The statistical fit indices for evaluating measurement invariance of the PECSS-CCS across gender groups are presented in Table 3. All models (M1, M2, M3, M4) show excellent fit to the indices, with CFI values consistently exceeding 0.960, SRMR values were close to 0.060, and RMSEA remaining below 0.050. Additionally, the changes in CFI, SRMR, and RMSEA (ΔCFI, ΔSRMR, and ΔRMSEA) are all under 0.010, indicating a strong model-data concordance. The multi-group invariance models across different gender groups demonstrate good invariance, as evidenced by all fit indices indicating an acceptable model-data fit. Therefore, the assumption of gender invariance for the PECSS-CCS is supported.

**Table 3:** Multigroup invariance model across gender.

Models	χ2	df	CFI	ΔCFI	TLI	SRMR	RMSEA (CI 90%)	ΔRMSEA
M1(configural)	798.657	568	0.965	–	0.959	0.049	0.041 (0.035–0.048)	–
M2(metric)	821.004	588	0.964	−0.001	0.960	0.054	0.041 (0.034–0.047)	0.000
M3(scalar)	835.799	608	0.965	+0.001	0.963	0.054	0.040 (0.033–0.046)	−0.001
M4(strict)	856.213	634	0.966	+0.001	0.965	0.057	0.039 (0.032–0.045)	−0.001

### Reliability test

The Cronbach’s *α* coefficient of each dimension of PECSS-CCS ranged from 0.83 to 0.92, the McDonald’s omega coefficient for the subscales ranged from 0.83 to 0.93, the CR value of each subscale of PECSS-CCS ranged from 0.84 to 0.93, all subscales’ test–retest reliability scored above 0.75, and the overall scale’s split-half reliability coefficient of 0.86, demonstrating that both subscales and the full scale met acceptable reliability standards (see Table 4).

**Table 4:** Validity and reliability of PECSS-CCS.

Subscale	CR	AVE	α	ω	Test–retest	Half-split	AU	CO	RE
TC	0.854	0.661	0.847	0.853	0.773	0.862	0.488^**^	0.436^**^	0.478^**^
TCA	0.926	0.677	0.924	0.925	0.884		0.375^**^	0.320^**^	0.433^**^
CE	0.857	0.602	0.853	0.855	0.752		0.554^**^	0.483^**^	0.491^**^
LA	0.841	0.575	0.827	0.833	0.782		0.433^**^	0.389^**^	0.496^**^
FA	0.901	0.645	0.899	0.897	0.854		0.380^**^	0.325^**^	0.491^**^
EX	0.904	0.702	0.901	0.907	0.838		0.433^**^	0.371^**^	0.495^**^

CR, composite reliability; AVE, average variance explained; α, Cronbach’s α; ω, McDonald omega coefficient; TC, teaching characteristics; TCA, teacher characteristics and attitudes; CE, course experience; LA, learning atmosphere; FA, facilities; EX, examination. AU, autonomy; CO, competence; RE, relatedness. ** p < 0.01.

### Validity test

The AVE value of each subscales were above 0.58, indicating good convergent validity, and Pearson correlation coefficient between the subscales of PECSS-CCS scores and BPNES scores ranging from 0.32 to 0.55 demonstrated strong criterion-related validity for the PECSS-CCS (see Table 4). These findings supported the criterion-related validity of the PECSS-CCS. Moreover, correlations among six factors ranged from 0.540 to 0.748, verified the previous hypothesis (see Figure 2). Overall, the results provided empirical support for the proposed factor structure, reliability, construct validity, criterion-related validity, and measurement invariance of the PECSS-CCS.

## Discussion

This study develops and validates a measurement scale for Chinese college students through internal consistency analysis, temporal stability analysis, construct validity analysis, convergent validity analysis, criterion-related validity analysis and measurement invariance analysis. The statistical results above revealed that this scale’s reliability and validity achieved a satisfactory psychometric standard. The formal PECSS-CCS comprises six dimensions namely teaching characteristics, teacher characteristics and attitudes, course experience, learning atmosphere, facilities and examinations.

The first dimension, teaching characteristics, measured whether college students were satisfied with their teaching content, method and schedule. The second dimension, teacher characteristics and attitudes, evaluates whether college students were satisfied with their teachers’ attitudes, morals, temperaments, presentation skills, guidance and communication skills. The third dimension, course experience, estimates whether college students were satisfied with their learning and improving their motor skills, physical fitness and knowledge. The fourth dimension, learning atmosphere, tested whether the college students were satisfied with their relationships with classmates, size and gender ratio of the class. The fifth dimension, facilities, examines whether college students were satisfied with the various conditions of sports equipment and venues. The sixth dimension, examination, assessed whether college students were satisfied with the exam’s content, format, grading standard and fairness.

Although some dimensions were similar to scales from previous studies, such as course experience (64), facilities (63), learning atmosphere and examination (60), teaching characteristics and teacher characteristics and attitudes were new dimensions. First, teaching characteristics involved the teaching content, method and schedule of the PE course, which was consistent with early research: (1) Designing course content was necessary and the delivery of the course content could achieve essential educational outcomes such as course satisfaction (61). (2) Methods such as providing various options, providing positive feedback and offering group activities will satisfy students’ psychological needs (84). (3) Schools should consider matching PE time allocation standards (85) and appropriate PE schedules should be adjusted to meet the requirements of different groups (86). Therefore, future research should consider enriching course content, innovating teaching methods and appropriately setting course units.

Second, the appearance of teacher characteristics and attitudes was consistent with previous studies which state that PE teachers must be proficient in correct techniques, tactics, rules, and etiquette (55, 62). Moreover, PE teachers’ beliefs and values had a notable influence on the PE curriculum (87) and their leadership styles could reinforce students’ learning motivation, efficiency, and satisfaction (88). Regarding teacher, scholars suggest that teacher training programs lay a solid foundation for PE qualities (89); however, some evidence has shown that current PE teacher training programs may fail to prepare PE instructors for their skills, knowledge, attitudes and fitness levels (90). Therefore, there is need for further research to solve this problem to further improve the quality of PE teachers and raise students’ satisfaction levels. Nonetheless, apart from teacher, school performance also plays an essential role in student satisfaction (91). For instance, colleges should provide more sports categories in PE courses to satisfy students’ different needs and interests (11).

Furthermore, beyond establishing the psychometric robustness of the PECSS-CCS, this study also highlights its broader relevance for physical education practice and research. This instrument enables administrators to identify deficiencies within PE courses, thereby informing reform strategies aimed at enhancing teaching effectiveness and student engagement. Moreover, the scale facilitates educators’ understanding of college students’ PE course satisfaction, allowing them to identify students’ actual physical and psychological needs. This understanding can subsequently inform interventions designed to improve student satisfaction and exercise attitudes toward PE. Additionally, the instrument provides researchers with a tool to examine the association between satisfaction with physical education courses and exercise behavior, for example, exploring how satisfaction experienced during PE courses facilitates improved health outcomes and adherence to future healthy lifestyle habits (92).

### Practical applications and implications for future practice and research

Recent research has highlighted the importance of using psychometrically sound assessment tools to inform evidence-based decision-making in physical education teaching, curriculum reform, and future research, as demonstrated by studies employing validated and reliable measures to examine key pedagogical variables (93). The PECSS-CCS provides meaningful applications for physical education teachers, administrators. For PE teachers, the multidimensional structure of the scale enables the identification of teaching-related factors influencing student satisfaction, thereby informing targeted adjustments to instructional strategies, course organization, and learning environments. For educational administrators, the PECSS-CCS serves as a standardized and psychometrically robust tool for monitoring PE course quality. Supporting evidence-based decisions in curriculum reform, teacher evaluation, and resource allocation by distinguishing sources of student dissatisfaction. From a public health perspective, students’ experiences and attitudes toward physical education are closely linked to broader psychological and behavioral health outcomes (94). Future research may apply the PECSS-CCS in longitudinal or intervention-based studies to investigate how improvements in specific satisfaction dimensions contribute to sustained physical activity engagement and broader public health outcomes.

### Limitations and future directions

Several limitations should be noted in this study. First, the participant cohort was exclusively recruited from public university, while private institutions were not included in the sampling frame. Thus, the statistical outcomes may yield divergent results if private universities are incorporated; subsequent research should validate the psychometric properties of the PECSS-CCS within this population. Second, although age, gender, and current grade level were recorded, other potentially influential background variables, including socioeconomic status, physical activity levels, and academic major, were not assessed. These factors may influence students’ motivation toward physical education and their satisfaction ratings. Future research should incorporate these variables to provide a more comprehensive interpretation of PE course satisfaction. Third, the cross-sectional methodological approach adopted herein precludes causal inference; follow-up investigations should undertake longitudinal tracking to assess temporal variations in college students’ physical education course satisfaction over time. Furthermore, all participants in this study came from the same geographic location; accordingly, future studies should expand samples to nationwide areas to evaluate the application of this scale. Lastly, we found that teacher characteristics and attitudes has the greatest impact on college students’ PE course satisfaction through the outcome of the cumulative variance contribution rate (95). Still, this phenomenon requires further investigation.

## Conclusion

Given the absence of specialized, validated assessment instruments for evaluating PE course satisfaction among Chinese college students in prior research, this study developed the 26-item PECSS-CCS. The investigation furnishes robust evidence supporting the validity and reliability of this measurement tool. The PECSS-CCS can be effectively utilized to assess PE course satisfaction within this population, establishing it as a practical assessment instrument for PE educators and researchers, consistent with recent research emphasizing validated and reliable measurement instruments to evaluate the effectiveness of physical education programs (96). Furthermore, this research offers valuable insights for college educators and administrators to enhance PE course design to foster improved satisfaction among Chinese college students, which is increasingly recognized as an important factor associated with students’ physical activity engagement and psychological well-being (93).

## Data Availability

The raw data supporting this study will be provided by the corresponding author upon reasonable request.

## References

[1] Alothman SA Al Baiz AA Alzaben AS Khan R Alamri AF Omer AB. Factors associated with lifestyle behaviors among university students—a cross-sectional study. Healthcare. (2024) 12:154. doi: 10.3390/healthcare12020154, 38255042 PMC10815065

[2] Poobalan AS Aucott LS Clarke A Smith WCS. Physical activity attitudes, intentions and behaviour among 18–25 year olds: a mixed method study. BMC Public Health. (2012) 12:640. doi: 10.1186/1471-2458-12-640, 22892291 PMC3490897

[3] Rodemann AE Arigo D. Subjective life expectancy among college students. Behav Med. (2018) 44:314–23. doi: 10.1080/08964289.2017.1378607, 28910192

[4] Zhang L Zhong T Dong K. University-based physical education as a structured temporal and spatial opportunity for shaping health-oriented lifestyles. Front Public Health. (2025) 13:1597480. doi: 10.3389/fpubh.2025.1597480, 40469591 PMC12133991

[5] Xiang J Peng F Jiao J Tan T Liu L Chen M . Health risk behaviors, depressive symptoms and suicidal ideation among college students: a latent class analysis in middle China. J Affect Disord. (2025) 375:205–13. doi: 10.1016/j.jad.2025.01.107, 39862987

[6] People’s Daily. As of 2020, 30% of Chinese college students failed to meet the physical fitness standards (2021). Available online at: https://www.peopleapp.com/column/30039198850-500004190915 (accessed April 24, 2021).

[7] Jin C Fan C Niu J. How physical exercise influences academic burnout among Chinese “double non” college students: the chain mediation role of mobile phone addiction and learning engagement. Front Psychol. (2024) 14:14. doi: 10.3389/fpsyg.2023.1289499, 38250123 PMC10797110

[8] Zhang J Zheng S Hu Z. The effect of physical exercise on depression in college students: the chain mediating role of self-concept and social support. Front Psychol. (2022) 13:13. doi: 10.3389/fpsyg.2022.841160, 35651580 PMC9150849

[9] Wang X Niu J Wang X Niu J. Linking exercise adherence to meaning in life through resilience and cognitive reappraisal: a sequential mediation model among Chinese university students. Behav Psychol. (2025) 33:44067. doi: 10.31083/BP44067

[10] Green K. Mission impossible? Reflecting upon the relationship between physical education, youth sport and lifelong participation. Sport Educ Soc. (2014) 19:357–75. doi: 10.1080/13573322.2012.683781

[11] Lackman J Smith ML McNeill EB. Freshman college students’ reasons for enrolling in and anticipated benefits from a basic college physical education activity course. Front Public Health. (2015) 3:162. doi: 10.3389/fpubh.2015.00162, 26157790 PMC4478376

[12] Carraro A Colangelo A Santi G Conti C Petrini M Gobbi E. An internet-supported continuing professional development training with secondary school physical education teachers: protocol for the physical education for moving (PE4MOVE) trial. Sustainability. (2022) 14:11579. doi: 10.3390/su141811579

[13] Han GS Han D Cho B Moon T-Y. Impact of satisfaction with physical education, general education courses on exercise adherence in university students. J Korea Acad Indus Coop Soc. (2010) 11:3380–9. doi: 10.5762/KAIS.2010.11.9.3380

[14] Kim NY Eun-Seok P. The relationship of participation motive on elective physical education courses in universities, class satisfaction, and exercise adherence. Korean Soc Sports Sci. (2014) 23:31–46.

[15] Richardson JTE. Instruments for obtaining student feedback: a review of the literature. Assess Eval High Educ. (2005) 30:387–415. doi: 10.1080/02602930500099193

[16] Sturm DJ Bachner J Haug S Demetriou Y. The German basic psychological needs satisfaction in physical education scale: adaption and multilevel validation in a sample of sixth-grade girls. Int J Environ Res Public Health. (2020) 17:1554. doi: 10.3390/ijerph17051554, 32121284 PMC7084701

[17] Trigueros R Mínguez LA González-Bernal JJ Aguilar-Parra JM Padilla D Álvarez JF. Validation of the satisfaction scale of basic psychological needs in physical education with the incorporation of the novelty in the Spanish context. Sustainability. (2019) 11:6250. doi: 10.3390/su11226250

[18] Vlachopoulos SP Katartzi ES Kontou MG. The basic psychological needs in physical education scale. J Teach Phys Educ. (2011) 30:263–80. doi: 10.1123/jtpe.30.3.263

[19] Tsangaridou N Pieroua M Charalambous CY. An analysis of content development in physical education: preschool teachers’ selection of instructional tasks. Eur Phys Educ Rev. (2023) 29:91–106. doi: 10.1177/1356336x221115376

[20] Cocca A Veulliet N Drenowatz C Wirnitzer K Greier K Ruedl G. Assessment of a novel instrument measuring perceived physical education teachers’ in-class skills. Behav Sci. (2023) 13:13. doi: 10.3390/bs13010042, 36661614 PMC9854623

[21] Porsanger L. Risk and safety management in physical education: teachers’ knowledge. Phys Educ Sport Pedagogy. (2023) 28:16–28. doi: 10.1080/17408989.2021.1934663

[22] Ward P Dervent F Devrilmez E Iserbyt P Kim I Ko B . Practice-based teacher education in physical education. J Teach Phys Educ. (2023) 42:442–51. doi: 10.1123/jtpe.2022-0047

[23] Schnitzius M Kirch A Spengler S Blaschke S Mess F. What makes a physical education teacher? Personal characteristics for physical education development. Br J Educ Psychol. (2021) 91:e12415. doi: 10.1111/bjep.12415, 33754352

[24] Black AE Deci EL. The effects of instructors’ autonomy support and students’ autonomous motivation on learning organic chemistry: a self-determination theory perspective. Sci Educ. (2000) 84:740–56. doi: 10.1002/1098-237X(200011)84:6<>3.0.CO;2-3

[25] Bergentoft H Annerstedt C Barker D Holmqvist M. Teachers’ actor-oriented transfer of movement pedagogy knowledge in physical education. Phys Educ Sport Pedagogy. (2024) 29:395–408. doi: 10.1080/17408989.2022.2083096

[26] Bracho-Amador CM Granero-Gallegos A Baena-Extremera A Lopez-Garcia GD. The effect of the motivational climate on satisfaction with physical education in secondary school education: mediation of teacher strategies in maintaining discipline. Behav Sci. (2023) 13:13. doi: 10.3390/bs13020178, 36829407 PMC9952606

[27] Viksi A Tilga H. Perceived physical education teachers’ controlling behaviour and students’ physical activity during leisure time-the dark side of the trans-contextual model of motivation. Behav Sci. (2022) 12:342. doi: 10.3390/bs12090342, 36135146 PMC9495742

[28] Sugiarto A Lee C-W Huruta AD. A systematic review of the sustainable campus concept. Behav Sci. (2022) 12:130. doi: 10.3390/bs12050130, 35621427 PMC9138111

[29] Jagerbrink V Glaser J Ostenberg AH. Extracurricular pulse activities in school: students’ attitudes and experiences. Int J Environ Res Public Health. (2022) 19:15051. doi: 10.3390/ijerph192215051, 36429770 PMC9691175

[30] Peng L Wei W Fan W Jin S Liu Y. Student experience and satisfaction in academic libraries: a comparative study among three universities in Wuhan. Buildings. (2022) 12:682. doi: 10.3390/buildings12050682

[31] Wilkins S Hazzam J Ireland JJ. Servicescape in transnational higher education: the effects of campus design, physical environment and facilities on student experience and satisfaction. J Mark High Educ. (2022) 34:992–1011. doi: 10.1080/08841241.2022.2139792

[32] Shin S Chiu W Lee H-W. For a better campus sporting experience: scale development and validation of the collegiate sportscape scale. J Hosp Leis Sport Tourism Educ. (2018) 22:22–30. doi: 10.1016/j.jhlste.2017.12.002

[33] Ferreira Silva RM Terra LF Valadao Fernandes M d S Silva Noll PRE de Abreu LC Noll M. Barriers to physical activity among full-time students: a case study during the COVID-19 pandemic. Sustainability. (2022) 14:11896. doi: 10.3390/su141911896, 41291439

[34] Raza A Pulakka A Hanson LLM Westerlund H Halonen JI. Distance to sports facilities and low frequency of exercise and obesity: a cross-sectional study. BMC Public Health. (2022) 22:2036. doi: 10.1186/s12889-022-14444-7, 36344975 PMC9641919

[35] Lee J Kim Y. A Meta-analysis of social ecological correlates of physical activity among Koreans. Percept Mot Skills. (2022) 129:1826–37. doi: 10.1177/00315125221126775, 36112888

[36] Mpalampa L Okoboi S Nabaggala SM Nanyonga RC. Factors associated with provision of physical activity in primary schools in Makindye division in Kampala, Uganda: a cross-sectional study. BMC Public Health. (2023) 23:23. doi: 10.1186/s12889-023-15216-7, 36774479 PMC9922171

[37] Chen W Hammond-Bennett A Hypnar A. Examination of motor skill competency in students: evidence-based physical education curriculum. BMC Public Health. (2017) 17:222. doi: 10.1186/s12889-017-4105-2, 28228116 PMC5322665

[38] Huhtiniemi M Saakslahti A Tolvanen A Watt A Jaakkola T. The relationships among motivational climate, perceived competence, physical performance, and affects during physical education fitness testing lessons. Eur Phys Educ Rev. (2022) 28:594–612. doi: 10.1177/1356336x211063568

[39] Simonton KL Mercier K Garn AC. Do fitness test performances predict students’ attitudes and emotions toward physical education? Phys Educ Sport Pedagogy. (2019) 24:549–64. doi: 10.1080/17408989.2019.1628932

[40] Keating XD Stephenson R Hodges M Zhang Y Chen LL. An analysis of Chinese preservice physical education teachers’ attitudes toward school-based fitness testing in physical education settings. Phys Educ Sport Pedagogy. (2021) 26:345–58. doi: 10.1080/17408989.2020.1806994

[41] Quennerstedt M Barker D Johansson A Korp P. Teaching with the test: using fitness tests to teach paradoxically in physical education. Eur Phys Educ Rev. (2025) 31:462–81. doi: 10.1177/1356336X241283796

[42] Vala R Valová M Litschmannová M Klimtová H. Sprinting abilities of year six students undergoing additional physical education classes. New Educ Rev. (2010) 22:165.

[43] Peng L Jin S Deng Y Gong Y. Students’ perceptions of active learning classrooms from an informal learning perspective: building a full-time sustainable learning environment in higher education. Sustainability. (2022) 14:8578. doi: 10.3390/su14148578

[44] Sato T Miller RT Delk DW. Secondary physical educators’ positioning of teaching English language learners at urban schools. Urban Educ. (2022) 57:814–41. doi: 10.1177/0042085918789747

[45] El-Jor C Rahi B Malhame MEK Mattar L Moussa S Zeeni N. Assessment of the world food Programme summer camps in Lebanon: a model of effective interventions for vulnerable adolescents. Br J Nutr. (2021) 125:1416–26. doi: 10.1017/s0007114520003682, 32943132

[46] Wang S Han C. The influence of learning styles on perception and preference of learning spaces in the university campus. Buildings. (2021) 11:572. doi: 10.3390/buildings11120572

[47] Ezeddine G Souissi N Masmoudi L Trabelsi K Puce L Clark CCT . The problem-solving method: efficacy for learning and motivation in the field of physical education. Front Psychol. (2023) 13:13. doi: 10.3389/fpsyg.2022.1041252, 36760899 PMC9905627

[48] Bos E Alinaghizadeh H Saarikoski M Kaila P. Factors associated with student learning processes in primary health care units: a questionnaire study. Nurse Educ Today. (2015) 35:170–5. doi: 10.1016/j.nedt.2014.09.012, 25456253

[49] Nepal B Taketomi K Ito YM Kohanawa M Kawabata H Tanaka M . Nepalese undergraduate nursing students’ perceptions of the clinical learning environment, supervision and nurse teachers: a questionnaire survey. Nurse Educ Today. (2016) 39:181–8. doi: 10.1016/j.nedt.2016.01.006, 27006054

[50] Pan Y-H. Relationships among teachers’ self-efficacy and students’ motivation, atmosphere, and satisfaction in physical education. J Teach Phys Educ. (2014) 33:68–92. doi: 10.1123/jtpe.2013-0069

[51] Standage M Duda JL Ntoumanis N. A test of self-determination theory in school physical education. Br J Educ Psychol. (2005) 75:411–33. doi: 10.1348/000709904x22359, 16238874

[52] Lyngstad I Bjerke O Lagestad P. “The teacher sees my absence, not my participation”. Pupils’ experiences of being seen by their teacher in physical education class. Sport Educ Soc. (2019) 24:147–57. doi: 10.1080/13573322.2017.1343713

[53] Lynch S Sargent J. Using the meaningful physical education features as a lens to view student experiences of democratic pedagogy in higher education. Phys Educ Sport Pedagog. (2020) 25:629–42. doi: 10.1080/17408989.2020.1779684

[54] Moore EWG Fry MD. Physical education students’ ownership, empowerment, and satisfaction with PE and physical activity. Res Q Exerc Sport. (2017) 88:468–78. doi: 10.1080/02701367.2017.1372557, 28967842

[55] Backman E Barker DM. Re-thinking pedagogical content knowledge for physical education teachers - implications for physical education teacher education. Phys Educ Sport Pedagog. (2020) 25:451–63. doi: 10.1080/17408989.2020.1734554

[56] Fang R Yang Z He Y Wang Y Zhang H. Effectiveness evaluation of physical education flipped classroom teaching based on knowledge construction. Mob Inf Syst. (2022) 2022:1507167. doi: 10.1155/2022/1507167

[57] Tan L Chen Q Wu J Li M Liu T. Optimizing physical education schedules for long-term health benefits. Front Public Health. (2025) 13:1555977. doi: 10.3389/fpubh.2025.1555977, 40589818 PMC12206874

[58] Grant JS Davis LL. Selection and use of content experts for instrument development. Res Nurs Health. (1997) 20:269. doi: 10.1002/(sici)1098-240x(199706)20:3<269::aid-nur9>3.3.co;2-3 9179180

[59] Shi Q. Reaserch on college students’s learning satisfacion in physical education class. J Cap Univ Phys Educ Sports. (2012) 24:42–45+53. doi: 10.14036/j.cnki.cn11-4513.2012.01.012

[60] Almeida LS Taveira M d C Peixoto F Silva JC Gouveia MJ. Academic domain satisfaction scale in Portuguese college students. Rev Iberoam Diagn Evaluacion-E Avaliacao Psicol. (2020) 1:93–101. doi: 10.21865/ridep54.1.08

[61] Eswaramoorthi V Kuan G Abdullah MR Majeed APPA Suppiah PK Musa RM. Design and validation of a virtual physical education and sport science-related course: a learner’s engagement approach. Int J Environ Res Public Health. (2022) 19:7636. doi: 10.3390/ijerph19137636, 35805306 PMC9265633

[62] Liu L Wang Y Wu T-J. Student satisfaction scale development and application for sport management in China. EURASIA J Math Sci Technol Educ. (2016) 13:1429–44. doi: 10.12973/eurasia.2017.00678a

[63] Rahmatpour P Peyrovi H Sharif NH. Development and psychometric evaluation of postgraduate nursing student academic satisfaction scale. Nurs Open. (2021) 8:1145–56. doi: 10.1002/nop2.727, 34482656 PMC8046041

[64] Costa PJC Inman RA Moreira PAS. The brief multidimensional students’ life satisfaction scale (BMSLSS): further evidence of factorial structure, reliability, and relations with other indicators of subjective wellbeing. Appl Res Qual Life. (2022) 17:3541–58. doi: 10.1007/s11482-022-10078-4

[65] Rubio DM Berg-Weger M Tebb SS Lee ES Rauch S. Objectifying content validity: conducting a content validity study in social work research. Soc Work Res. (2003) 27:94–104. doi: 10.1093/swr/27.2.94

[66] Sireci S Faulkner-Bond M. Validity evidence based on test content. Psicothema. (2014) 1:100–7. doi: 10.7334/psicothema2013.256, 24444737

[67] Bayne GA Inan FA. Development of the online course overload indicator and the student mental fatigue survey. Int Rev Res Open Distrib Learn. (2022) 23:74–92. doi: 10.19173/irrodl.v23i4.6223

[68] Phakiti A. Confirmatory factor analysis and structural equation modeling. In: Phakiti A De Costa P Plonsky L Starfield S editors. The Palgrave handbook of applied linguistics research methodology. London: Palgrave Macmillan. (2018) 459–500.

[69] Byrne BM. Structural equation modeling with AMOS: Basic concepts, applications, and programming, second edition. 2nd ed. New York: Routledge (2013). 416 p.

[70] Selig J Card NA Little T. Latent variable structural equation modeling in cross-cultural research: multigroup and multilevel approaches. In: van de Vijver FJR Van Hemert DA Poortinga YH editors. Multilevel analysis of individuals and cultures. New York: Taylor & Francis Group/Lawrence Erlbaum Associates (2008) 93–119.

[71] Vlachopoulos SP Michailidou S. Development and initial validation of a measure of autonomy, competence, and relatedness in exercise: the basic psychological needs in exercise scale. Meas Phys Educ Exerc Sci. (2006) 10:179–201. doi: 10.1207/s15327841mpee1003_4

[72] Baena-Extremera A Gómez-López M Granero-Gallegos A Martínez-Molina M. Modelo de predicción de la satisfacción y diversión en Educación Física a partir de la autonomía y el clima motivacional. Univ Psychol. (2016) 15:39. doi: 10.11144/Javeriana.upsy15-2.mpsd

[73] Liu JD Chung PK Duan Y. Validity and reliability of the Chinese translation of basic psychological needs in exercise scale. Eur J Psychol Assess. (2013) 29:51–7. doi: 10.1027/1015-5759/a000120

[74] Al Ansari A Strachan K Hashim S Otoom S. Analysis of psychometric properties of the modified SETQ tool in undergraduate medical education. BMC Med Educ. (2017) 17:56. doi: 10.1186/s12909-017-0893-4, 28302151 PMC5356325

[75] Kaiser HF. The application of electronic computers to factor analysis. Educ Psychol Meas. (1960) 20:141–51. doi: 10.1177/001316446002000116

[76] Hair JF Jr, Anderson RE Babin BJ Black WC. Multivariate data analysis: A global perspective. 7th ed. Upper Saddle River (N.J.): Pearson Education. (2010). Available online at: http://lib.ugent.be/catalog/rug01:001321386.

[77] Maskey R Fei J Nguyen H-O. Use of exploratory factor analysis in maritime research. Asian J Shipp Logist. (2018) 34:91–111. doi: 10.1016/j.ajsl.2018.06.006

[78] Schreiber JB Nora A Stage FK Barlow EA King J. Reporting structural equation modeling and confirmatory factor analysis results: a review. J Educ Res. (2006) 99:323–38. doi: 10.3200/JOER.99.6.323-338

[79] Chen FF. Sensitivity of goodness of fit indexes to lack of measurement invariance. Struct Equ Model Multidiscip J. (2007) 14:464–504. doi: 10.1080/10705510701301834

[80] Kalkbrenner MT. Choosing between Cronbach’s coefficient alpha, McDonald’s coefficient omega, and coefficient *H*: confidence intervals and the advantages and drawbacks of interpretive guidelines. Meas Eval Couns Dev. (2024) 57:93–105. doi: 10.1080/07481756.2023.2283637

[81] Mirhosseini S Ameri F Rahmani H Sharif-Nia H Fazel G Khajeh M . Psychometric assessment of the persian version of the study anxiety questionnaire in medical sciences students. BMC Med Educ. (2024) 24:1517. doi: 10.1186/s12909-024-06528-2, 39716142 PMC11664849

[82] Nunnally JC Bernstein IH. Psychometric theory (3rd ed.). New York: McGraw-Hill (1994).

[83] Tan W Chen J Lu S Liu C Luo Q Ma Y . Psychometric evaluation of the Chinese version of the academic resilience Scale-30 (C-ARS-30) in college students. Front Psychol. (2024) 15:1276618. doi: 10.3389/fpsyg.2024.1276618, 39171240 PMC11335623

[84] Chu TL Zhang T Cheung HY. The roles of need-supportive social environments in university physical education courses. Int J Sport Exerc Psychol. (2017) 17:212–31. doi: 10.1080/1612197x.2017.1339727

[85] Kahan D McKenzie TL. Correlates of private secondary schools meeting physical education guidelines. J Sch Health. (2018) 88:508–15. doi: 10.1111/josh.12633, 29864205

[86] Kahan D McKenzie TL. California districts and schools underutilize websites to demonstrate compliance to a physical education lawsuit. Res Q Exerc Sport. (2019) 90:712–9. doi: 10.1080/02701367.2019.1623855, 31282787

[87] Capel S. Value orientations of student physical education teachers learning to teach on school-based initial teacher education courses in England. Eur Phys Educ Rev. (2016) 22:167–84. doi: 10.1177/1356336x15596984

[88] Jia Z-R Jiang Z. Effects of physical education teachers’ leadership styles and classroom climate on learning motivation for basketball course. EURASIA J Math Sci Technol Educ. (2018) 14:1351–7. doi: 10.29333/ejmste/81296

[89] Yildizer G Munusturlar S. Differences in perceived physical literacy between teachers delivering physical education in schools: classroom teachers vs physical education teachers. Phys Educ Sport Pedagogy. (2021) 27:626–39. doi: 10.1080/17408989.2021.1932784

[90] Bulger SM Mohr DJ Carson LM Wiegand RL. Infusing health-related physical fitness in physical education teacher education. Quest. (2001) 53:403–17. doi: 10.1080/00336297.2001.10491755

[91] Biswas K Bose S Chang M Shams S. Determinants and consequences of student satisfaction in Australian universities: evidence from QILT surveys. Account Finance. (2022) 00:1–30. doi: 10.1111/acfi.12930

[92] Rojo-Ramos J González-Becerra MJ Gómez-Paniagua S Adsuar JC. Satisfaction with physical activity among students in the last cycle of primary education in Extremadura. Int J Environ Res Public Health. (2022) 19:6702. doi: 10.3390/ijerph19116702, 35682286 PMC9180547

[93] Chen Z Tian Y Li M Yang S. Personality traits and Chinese college students’ satisfaction with physical education classes: the mediating role of trait fluency and the moderating role of physical education class difficulty. Front Psychol. (2023) 14:14. doi: 10.3389/fpsyg.2023.1270089, 38173850 PMC10762447

[94] Li X Cui L Shen Q-Q Luo R Liu M. Relationship between Chinese college students’ attitude to physical exercise and psychological capital: the mediating effects of self-control and gender. Front Public Health. (2024) 12:1443489. doi: 10.3389/fpubh.2024.1443489, 39600401 PMC11588726

[95] Lv Y Zhang X Zhang P Wang H Ma Q Tao X. Comparison between voltammetric detection methods for abalone-flavoring liquid. Open Life Sci. (2021) 16:354–61. doi: 10.1515/biol-2021-0035, 33954255 PMC8051168

[96] Qin L King Yan Ho W Xie Y Wang J Cheng L Liu Z . The development and validation of a scale to assess perception of physical education among university students in China. SAGE Open. (2024) 14:21582440241228910. doi: 10.1177/21582440241228910

